# The Canal of Joseph

**Published:** 1890-05

**Authors:** 


### THE CANAL OF JOSEPH.

How many of the engineering works of the nineteenth century will there be in existence in the year 6000? Very few, we fear, says *Engineering*, and still less those that will continue in the far off age to serve a useful purpose. Yet there is at least one great undertaking conceived and executed by an engineer which, during the space of 4,000 years, has never ceased its office, on which the life of a fertile province absolutely depends to-day. We refer to the Bahr Joussuf—the canal of Joseph—built, according to tradition, by the son of Jacob, and which constitutes not the least of the many blessings he conferred on Egypt during the years of his prosperous rule. This canal took its rise from the Nile at Asiut, and ran nearly parallel with it for nearly 250 miles, creeping along under the western cliffs of the Nile valley, with many a bend and winding, until at length it gained an eminence, as compared with the river bed, which enabled it to turn westward through a narrower pass and enter a district which was otherwise shut off from the fertilizing floods on which all vegetation in Egypt depend.

The northern end stood seventeen feet above low Nile, while at the southern end it was at an equal elevation with the river. Through this cut ran a perennial stream, which watered a province named the Fayoum, endowing it with fertility and supporting a large population. In the time of the annual flood a great part of the canal was under water, and the river's current would rush in a more direct course into the pass, carrying into it the rich silt which takes the place of manure and keeps the soil in a state of constant productiveness.

All this, with the exception of the tradition that Joseph built it, can be verified to-day, and it is not mere supposition or rumor.

Many accounts have been written by Greek and Roman historians, such as Herodotus, Strabo, Mutianus, and Pliny, and repeated in monkish legends or portrayed in the maps of the middle ages, which agreed with the folk lore of the district. These tales explained that the canal dug by the ancient Israelites served to carry the surplus waters of the Nile into an extensive lake lying south of the Fayoum, and so large that it not only modified the climate, tempering the arid winds of the desert and converting them into the balmy airs which nourished the vines and the olives into a fullness and fragrance unknown in any part of the country, but also added to the food supply such immense quantities of fish that the royal prerogative of the right of piscatory at the great weir was valued at $250,000 annually. This lake was said to be 450 miles round, and to be navigated by a fleet of vessels, while the whole circumference was the scene of industry and prosperity.

